# Efficacy of Almonertinib Versus Osimertinib as the First‐Line Treatment for Non‐Small Cell Lung Cancer With EGFR L858R Mutation and Prognostic Analysis: A Retrospective Comparative Cohort Study

**DOI:** 10.1002/cam4.71422

**Published:** 2025-11-27

**Authors:** Xiujing Yao, Ruyue Li, Xue Dong, Ying Li, Yintao Li

**Affiliations:** ^1^ School of Clinical Medicine Shandong Second Medical University Weifang Shandong China; ^2^ Department of Respiratory Oncology, Shandong Cancer Hospital and Institute Shandong First Medical University and Shandong Academy of Medical Sciences Jinan Shandong China

**Keywords:** almonertinib, brain metastasis, efficacy, EGFR L858R mutation, osimertinib

## Abstract

**Objectives:**

Epidermal growth factor receptor (EGFR)–specific tyrosine kinase inhibitors show less efficacy against the EGFR L858R mutation than EGFR 19del, but no current head‐to‐head clinical trials have been performed comparing the efficacy of almonertinib and osimertinib. Therefore, our study compared the efficacy of these drugs against the EGFR L858R mutation.

**Materials and Methods:**

A total of 200 patients with non‐small cell lung cancer harboring the EGFR L858R mutation were enrolled. Among these patients, 121 received 80 mg of osimertinib, while the other 79 received 110 mg of almonertinib once daily. The primary end point was progression‐free survival (PFS). The secondary end points were continued response rate and safety.

**Results:**

The median PFS was 18.5 months (95% confidence interval [CI] 16.1–22.5) for osimertinib and 19.4 months (95% CI 13.8–NA) for almonertinib, with a hazard ratio (HR) of 0.92 (95% CI 0.62–1.73), *p* = 0.69. Forest plots of subgroup analyses showed no significant difference in the median PFS between the almonertinib and osimertinib groups across the subgroups. Osimertinib and almonertinib demonstrated good efficacy in the treatment of patients with brain metastases. The median PFS was 18.6 months (95% CI 15.6–22.8) for patients with brain metastases and 17.1 months (95% CI 14.1–28.6) for those without brain metastases, *p* = 0.89. Patients with programmed cell death ligand 1 (PD‐L1) expression between 1% and 49% and PD‐L1 expression < 1% showed no significant differences for their median PFS. The continued response rates between almonertinib and osimertinib were comparable. The differences between almonertinib and osimertinib were minimal.

**Conclusion:**

Both almonertinib and osimertinib demonstrated good efficacy in patients with brain metastases, and PD‐L1 expression was not associated with the prognosis of EGFR L858R mutant NSCLC. Finally, no significant difference between osimertinib and almonertinib for the treatment of patients with EGFR L858R mutations was observed. Both options remain viable for these patients.

## 1 Introduction

Lung cancer is the leading cause of cancer‐related death worldwide [1, 2]. Epidermal growth factor receptor (EGFR) mutations account for 15%–25% of non‐small cell lung cancer (NSCLC) cases [3]. EGFR mutations in the tyrosine kinase (TK) domain are more common in never‐smokers than in smokers (51% versus 10%), in adenocarcinoma than in other histological types (40% versus 3%), in patients of East Asian ethnicity than in those of other ethnicities (30% versus 8%), and in females than in males (42% versus 14%) [4]. Exon 19 deletion (19del) of EGFR is the most prevalent alteration in NSCLC, followed by L858R mutation in exon 21 L858R [5, 6].

Osimertinib has been approved as a first‐line treatment [7] in most countries, including the United States and the European Union, for patients with locally advanced or metastatic NSCLC [8, 9] harboring an EGFR exon 19 deletion or exon 21 L858R mutation (activating EGFR mutations) [10]. Osimertinib demonstrated superior efficacy compared with first‐ and second‐generation EGFR‐tyrosine kinase inhibitors (TKIs). In the FLAURA clinical trial, the median progression‐free survival (mPFS) reached 18.9 months in the osimertinib group, compared to 10.2 months in the control group, extending survival by 8.7 months. The hazard ratio (HR) of 0.46 represented a 54% reduction in the risk of progression or death. Compared with the control group, osimertinib reduced the risk of patient death by 20% [11, 12]. After a median follow‐up of 12.3 months in real‐world studies, the median time to treatment discontinuation was 25.3 months; the median progression‐free survival (mPFS) was 18.9 months. The median follow‐up times for the osimertinib group and the first‐generation TKI group were 35.8 months and 27.0 months, respectively. The median overall survival (OS) times were 38.6 months and 31.8 months, respectively. The risk of disease‐related death decreased by 20% (HR = 0.80; 95% confidence interval (CI): 0.64–1.00; *p* = 0.046) [13]. Although osimertinib has shown good efficacy in treating patients with NSCLC harboring the EGFR L858R mutation, its effectiveness is typically more pronounced in patients harboring the EGFR 19del mutation [6, 11].

Almonertinib was the first domestically developed third‐generation EGFR–TKI in China. It is a novel, irreversible, and structurally predictive third‐generation EGFR–TKI that selectively inhibits both sensitive and resistant EGFR mutations [14, 15]. In the AENEAS study, the mPFS in the almonertinib group reached 19.3 months, which was extended by 9.4 months compared to the gefitinib group (9.9 months). The risk of disease progression or death was reduced by approximately 54% (HR = 0.463, 95% CI: 0.359–0.596, *p* < 0.0001). Subgroup analysis revealed that almonertinib significantly reduced the risk of brain metastasis progression by 62% (HR = 0.38) [16, 17]. Based on the results of the AENEAS study, almonertinib was approved in China for the treatment of advanced NSCLC with EGFR mutations. Unlike osimertinib, which has received global approval, almonertinib is currently approved only in China [18]. Almonertinib and osimertinib lack head‐to‐head clinical trials for direct comparison; therefore, current clinical trial efficacy data do not suggest which one, almonertinib or osimertinib, has better therapeutic efficacy. However, in terms of the treatment efficacy of third‐generation EGFR–TKIs, 19del mutations show a higher objective response rate (ORR) than that of L858R mutations [19, 20, 21, 22]. Without considering other accompanying mutations, 19del mutations also exhibit better treatment efficacy [21, 23].

At present, clinical trials have not yet indicated which one, almonertinib or osimertinib, has better therapeutic efficacy in EGFR 21 L858R mutations. However, a comparison of the efficacy of almonertinib and osimertinib in patients with NSCLC harboring the L858R mutation is of great interest. Therefore, this study aimed to retrospectively compare the clinical efficacy of almonertinib and osimertinib in these patients with the aim of providing more accurate treatment choices for clinical practice.

## 2 Materials and Methods

### 2.1 Study Population

**FIGURE 1:**
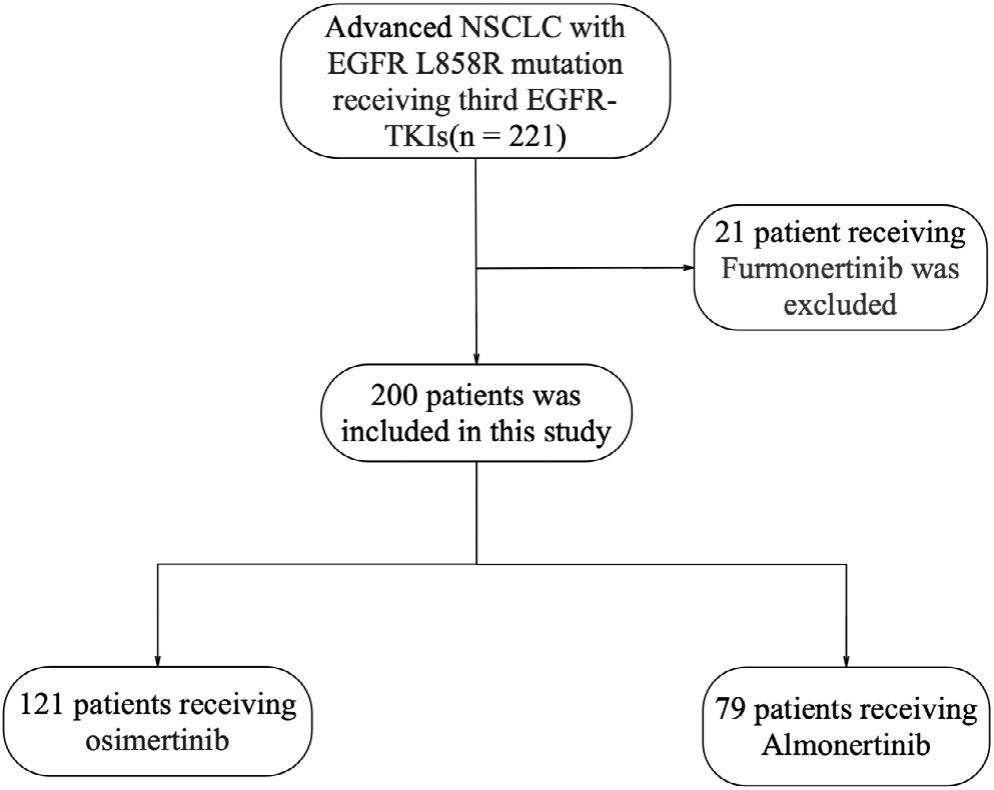
Flow chart of the search strategy and study selection.

This retrospective clinical study concerns the efficacy of almonertinib and osimertinib in patients with advanced NSCLC. Two hundred patients with NSCLC harboring the EGFR L858R mutation were enrolled at the Shandong Cancer Hospital and Institute, between January 1, 2020, and February 1, 2023. Among these patients, 121 received 80 mg of osimertinib, while the other 79 took 110 mg of almonertinib once daily. The inclusion criteria were as follows: (1) next‐generation sequencing (NGS) testing that confirmed the presence of the EGFR L858R mutation, (2) clinical stage IIIB or IV NSCLC, (3) switching to either osimertinib or ameliorating after experiencing severe adverse reactions to earlier EGFR–TKIs, and (4) experiencing recurrence after surgical treatment, with clinical staging at stage IV. Exclusion criteria included: (1) receiving an earlier generation of EGFR‐TKI therapy as initial treatment until recurrence, (2) targeted therapy combined with chemotherapy or antiangiogenesis treatment, (3) death from noncancer causes, and (4) incomplete documentation of imaging or clinicopathological information and lack of follow‐up visits. Ultimately, 200 patients with stage IIIB or IV NSCLC and EGFR L858R mutations were eligible for this study and assigned to receive osimertinib (*n* = 121) or almonertinib (*n* = 79). A flowchart of patient enrollment is shown in Figure 1. The ethics committee of the Shandong Cancer Hospital and Institute approved this study. The International Union against Cancer and the American Joint Committee on Cancer 9th edition TNM staging systems were used. This study was conducted in accordance with the principles of the Declaration of Helsinki and approved by the tumor hospital Shandong Cancer Hospital and Institute.

### 2.2 Data Collection

Clinical data of all eligible patients were retrospectively collected from medical records. Patient demographics included gender, age, and smoking habits. Clinical characteristics comprised Eastern Cooperative Oncology Group (ECOG) performance status, clinical stage at diagnosis, brain metastasis status, liver metastasis status, bone metastasis status, adrenal metastases status, and history of surgery. Molecular characteristics included programmed cell death ligand 1 (PD‐L1) expression and TP53 mutation status. Adverse events (AEs) were retrospectively extracted from patients' electronic medical records and follow‐up notes. AEs were graded according to the National Cancer Institute Common Terminology Criteria for AEs, version 5.0.

### 2.3 Study End points and Follow‐Up

The primary end point, PFS, and clinical outcomes were assessed for each patient. The PFS was evaluated from the first day of almonertinib or osimertinib administration until relapse. All enrolled patients underwent regular outpatient reviews and telephone follow‐ups after admission, with regular physical examinations and chest computed tomography (CT) during follow‐up. For patients whose last case record in the case system was recorded more than one month before the cutoff time of this study, telephone follow‐up was used to complete the collection of patients' clinical data and to establish a database for statistical analysis. They were asked for details about their disease progression and survival. Follow‐up ended on January 10, 2024, and the median duration of follow‐up for all patients was 19.3 months (range, 0.7–40.3 months).

### 2.4 EGFR Genotyping

Tumor tissues were obtained via puncture biopsy or fiberoptic bronchoscopy. Both formalin‐fixed paraffin‐embedded (FFPE) and freshly frozen samples were reviewed by pathologists to ensure ≥ 20% tumor content and sufficient material for analysis. Subsequently, amplification refractory mutation system polymerase chain reaction (ARMS‐PCR) was performed to amplify the EGFR gene, and mutation detection was conducted using the ADx EGFR Mutation Detection Kit [24]. In addition, molecular profiling was carried out using next‐generation sequencing (NGS). The detailed detection procedures have been described in previous reports [25, 26].

### 2.5 Statistical Analysis

Continuous variables were summarized as mean ± standard deviation (SD) if normally distributed, or as median and interquartile range (IQR) if non‐normally distributed. Normality of continuous variables was assessed using the Shapiro–Wilk test. Differences between continuous variables were evaluated using Student's *t*‐test for normally distributed data or the Wilcoxon rank‐sum test for non‐normally distributed data. Categorical variables were compared using Pearson's chi‐square test or Fisher's exact test as appropriate. Survival curves were estimated using the Kaplan–Meier method, and differences between groups were assessed using the log‐rank test. Univariate Cox proportional hazards regression models were used to examine potential risk factors for PFS, with covariates including age (continuous variable), gender, ECOG performance, TP53 mutation, and metastatic status (categorical variables). HR with 95% CI was reported. To control for multiple testing, *q*‐values were calculated using the Benjamini–Hochberg procedure to control the false discovery rate (FDR). All *p*‐values were two‐sided, and *p*‐value < 0.05 was considered statistically significant. All analyses and figures were performed using R software (version 4.0.3) with the following packages: survival (v3.5‐3), survminer (v0.4.9).

## 3 Results

### 3.1 Patient Characteristics

The baseline characteristics of the 200 patients with NSCLC included in this study are shown in Table 1. The median age for the osimertinib group (*n* = 121) was 61.93 ± 8.99 years, while the median age for the almonertinib group (*n* = 79) was 62.82 ± 9.34 years. Differences between the two groups were not significant (Table 1). At the same time, univariate Cox regression analysis showed no variables reached statistical significance for PFS, while multivariate analysis indicated that bone metastasis was significantly associated with PFS (*p* = 0.032) (Table 2).

**TABLE 1:** Demographic of the patients at baseline.

Patients' characteristic			
Characteristic	Osimertinib	Almonertinib	*p* ^b^
	*n* = 121^a^	*n* = 79^a^	
Age			
Mean (SD)	61.93 ± 8.99	62.82 ± 9.34	0.505
Gender			
Female	77 (64%)	47 (59%)	0.556
Male	44 (36%)	32 (41%)	
ECOG			
0	93 (77%)	60 (76%)	0.918
1	27 (22%)	19 (24%)	
2	1 (0.8%)	0 (0%)	
Smoker			
Yes	15 (12%)	17 (22%)	0.114
No	106 (88%)	62 (78%)	
TP53 mutations			
Yes	3 (2.5%)	3 (3.8%)	0.682
No	118 (98%)	76 (96%)	
Histopathology			
Adenocarcinoma	119 (98%)	78 (99%)	
Adenosquamous carcinoma	1 (0.8%)	0 (0%)	
Squamous cell carcinoma	0 (0%)	1 (1.3%)	
Mucoepidermoid carcinoma	1 (0.8%)	0 (0%)	
PD‐L1			
≥ 50%	14 (12%)	3 (3.8%)	0.251
1%–49%	23 (19%)	19 (24%)	
< 1%	30 (25%)	21 (27%)	
Unknown	54 (45%)	36 (46%)	
Stage			
IIIB	1 (0.8%)	5 (6.3%)	0.036
IV	120 (99%)	74 (94%)	
Surgery			
Yes	7 (5.8%)	3 (3.8%)	0.743
No	114 (94%)	76 (96%)	
Brain metastasis			
Yes	64 (53%)	35 (44%)	0.25
No	57 (47%)	44 (56%)	
Bone metastasis			
Yes	66 (55%)	39 (49%)	0.563
No	55 (45%)	40 (51%)	
Liver metastasis			
Yes	8 (6.6%)	7 (8.9%)	0.59
No	113 (93%)	72 (91%)	
Adrenal metastasis			
Yes	11 (9.1%)	3 (3.8%)	0.256
No	110 (91%)	76 (96%)	

*Note:* Age is presented as mean ± standard deviation.

Abbreviations: ECOG, Eastern Cooperative Oncology Group; PD‐L1, programmed cell death ligand 1.

^a^ *n* (%).

^b^ Welch two sample *t*‐test: Fisher's exact test.

**TABLE 2:** Univariate and multivariate analyses of clinical variables for progression‐free survival.

Variable	Univariate analysis		Multivariate analysis	
	Hazard ratio (95% CI)	*p*	Hazard ratio (95% CI)	*p*
Smoker (yes vs. no)	0.824 (0.483–1.407)	0.478	0.892 (0.471–1.689)	0.726
Gender (female vs. male)	1.168 (0.789–1.730)	0.437	1.177 (0.750–1.846)	0.478
Age	0.996 (0.974–1.018)	0.723	0.987 (0.964–1.011)	0.295
ECOG (0 vs. 1)	1.366 (0.881–2.118)	0.164	0.751 (0.474–1.191)	0.223
PD‐L1 (≥ 50% vs. 1%–49%)	1.041 (0.464–2.334)	0.923	1.160 (0.491–2.739)	0.735
PD‐L1 (≥ 50% vs. < 1%)	1.126 (0.512–2.479)	0.768	0.920 (0.396–2.138)	0.846
Brain metastasis (yes vs. no)	0.973 (0.665–1.422)	0.886	1.007 (0.677–1.499)	0.971
Bone metastasis (yes vs. no)	0.686 (0.465–1.014)	0.059	0.638 (0.422–0.963)	0.032
Liver metastasis (yes vs. no)	0.547 (0.292–1.026)	0.06	0.504 (0.253–1.003)	0.051
Adrenal metastasis (yes vs. no)	0.695 (0.337–1.433)	0.324	0.738 (0.343–1.588)	0.437
Group (osimertinib vs. almonertinib)	0.922 (0.622–1.367)	0.686	0.895 (0.590–1.356)	0.6

Abbreviation: ECOG, Eastern Cooperative Oncology Group.

### 3.2 Therapeutic Effect of Osimertinib Versus Almonertinib and Subgroup Analysis

**FIGURE 2:**
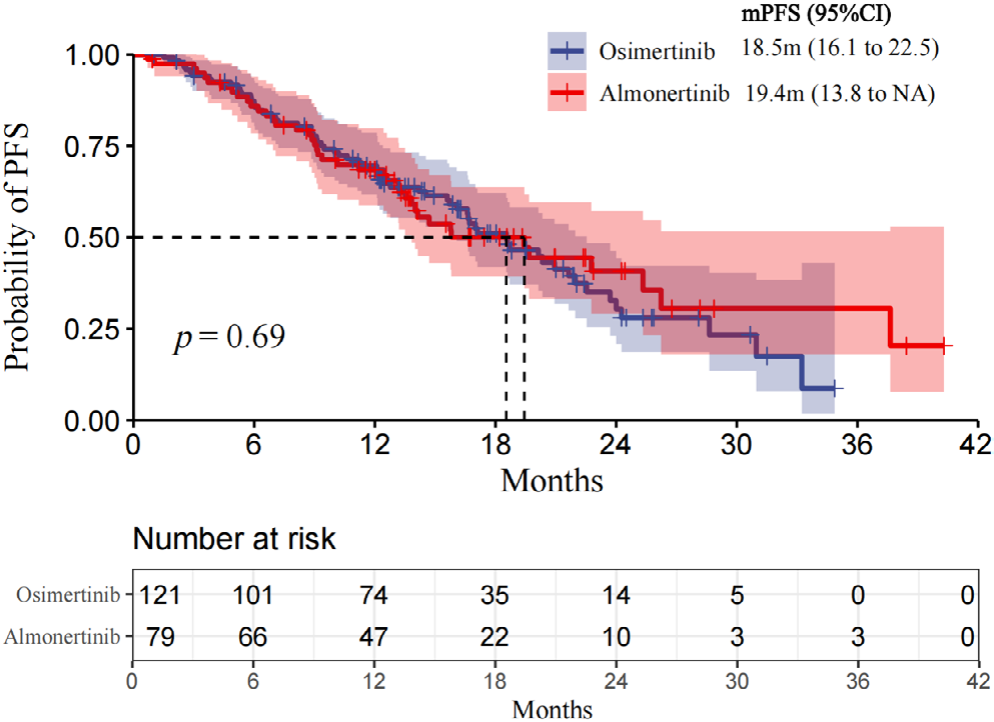
Osimertinib versus almonertinib for progression‐free survival. The Kaplan–Meier estimate of progression‐free survival is shown. In the full analysis set. The censored data are indicated by tick marks. Data from patients who did not show progression at the time of analysis were censored based on the last recorded date on which the patient was known to be progressing.

Kaplan–Meier analysis showed no significant difference in PFS between almonertinib and osimertinib. The median PFS was 18.5 months [95% CI, 16.1–22.5] for osimertinib and 19.4 months [95% CI, 13.8–NA] for almonertinib, with a HR of 0.92 [95% CI, 0.62–1.73], *p* = 0.69, (Figure 2). At 12 months, the continued response rates for osimertinib and almonertinib were 93.4% versus 94.8%; at 18 months, 87.8% versus 81.4%; and at 24 months, 74.8% versus 70.9%, indicating that the continued response rates of the two agents were comparable (Table 3).

**TABLE 3:** Continued response rates at 12, 18, and 24 months.

Continued response (95% CI)—%	Osimertinib	Almonertinib
At 12 months	93.4 (89.1–97.9)	94.8 (90–97.9)
At 18 months	87.8 (81.7–94.4)	81.4 (72.6–91.2)
At 24 months	74.8 (65.8–85)	70.9 (59.5–87.4)

**FIGURE 3:**
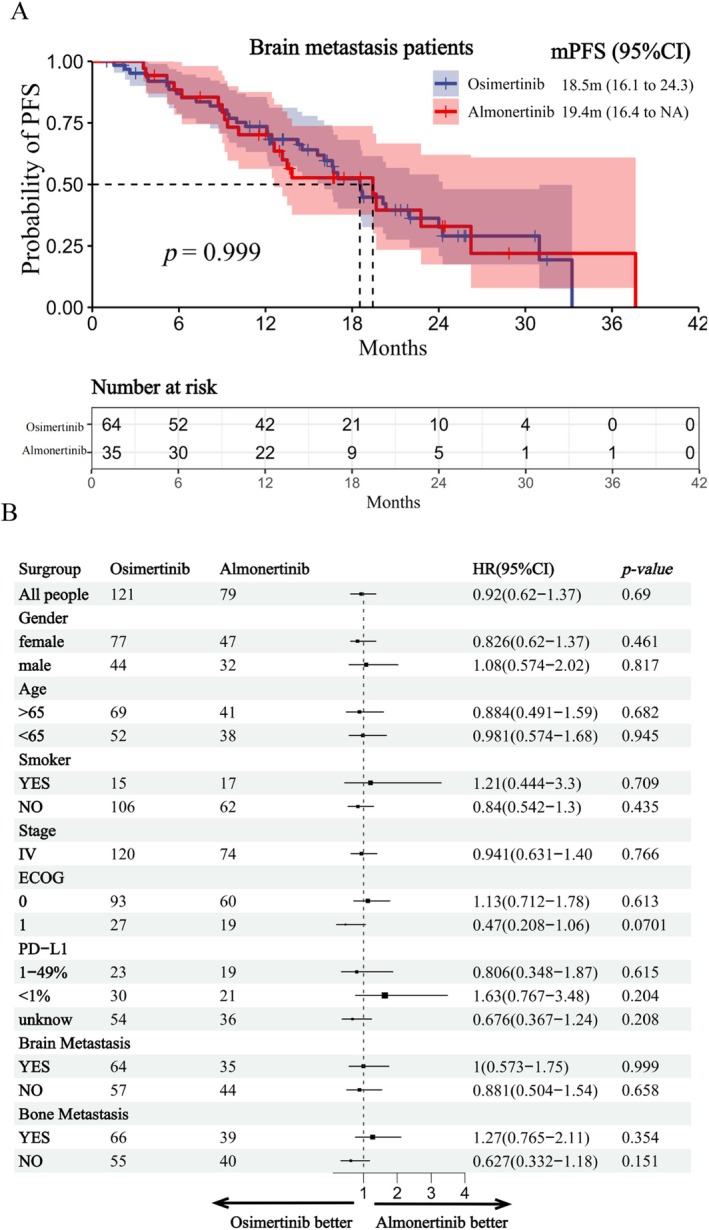
Subgroup analyses of progression‐free survival of almonertinib versus osimertinib. (A) Comparison of almonertinib and osimertinib in patients with brain metastases. Kaplan–Meier curves were used to describe progression‐free survival. (B) Progression‐free survival by subgroup in the full analysis set; ECOG PS, Eastern Cooperative Oncology Group performance status; PD‐L1, programmed cell death ligand 1; HR, hazard ratio. A forest plot of the subgroup analyses was constructed using a Cox proportional hazards model that included the group, subgroup covariate of interest, and treatment‐by‐subgroup interactions. Population analyses were performed using stratified log‐rank tests.

In patients with brain metastases, no significant difference in the efficacy between almonertinib and osimertinib was observed. The median PFS was 18.5 months [95% CI, 16.1–24.3] for osimertinib and 19.4 months [95% CI 16.4–NA] for almonertinib, with a HR of 0.92 [95% CI, 0.573–1.75], *p* = 0.999 (Figure 3A). Additionally, forest plots of subgroup analyses showed no significant differences in the median PFS between the almonertinib and osimertinib groups across the subgroups defined by baseline gender, age, smoking status, clinical stage, ECOG status (ECOG‐PS 0 and ECOG‐PS 1), PD‐L1 expression status, and presence of brain and bone metastases (Figure 3B).

### 3.3 Osimertinib and Almonertinib Exhibit Good Efficacy in the Treatment of Patients With Brain Metastases

**FIGURE 4:**
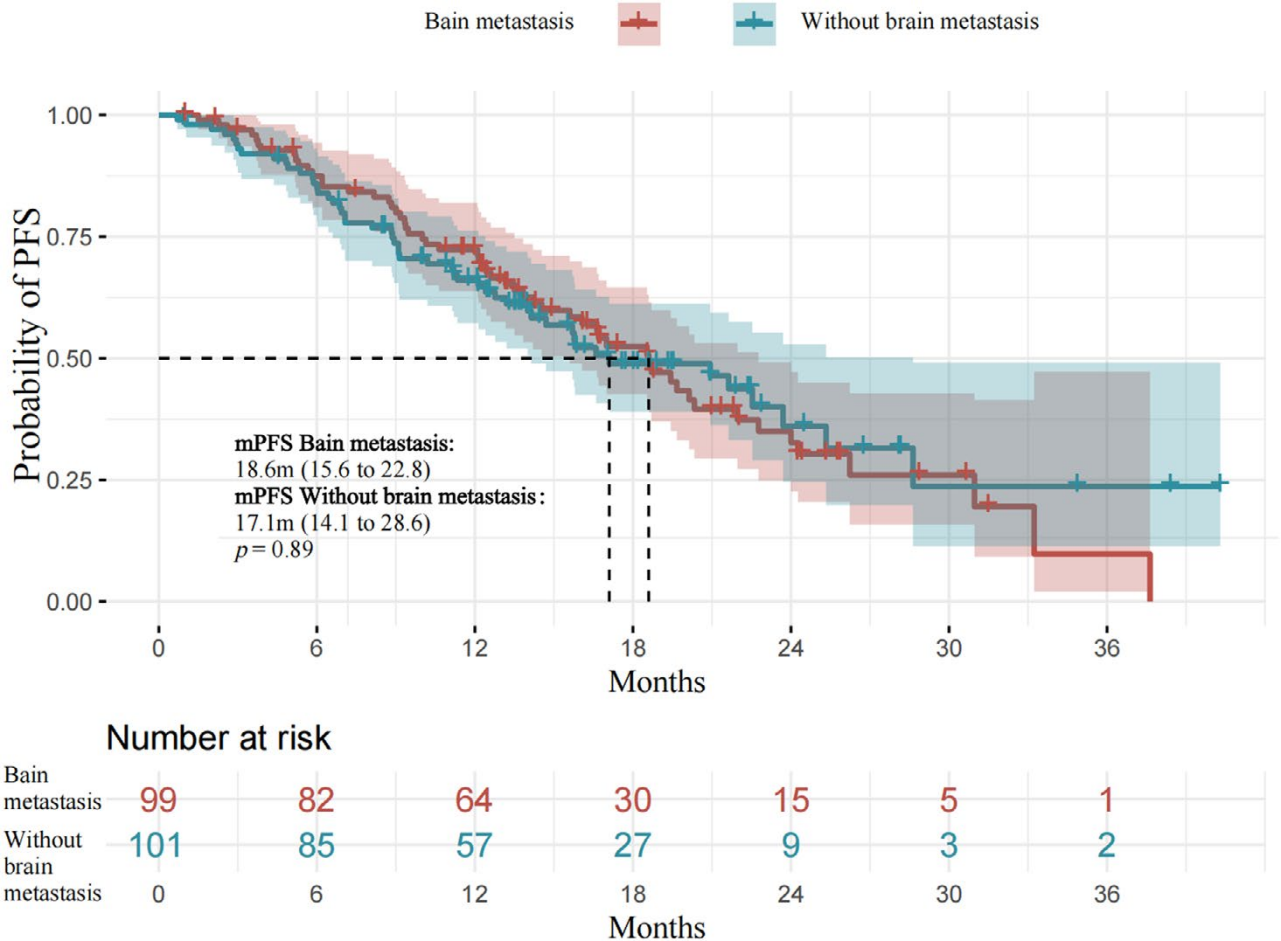
Kaplan–Meier curves comparing the progression‐free survival of patients with or without brain metastases. In patients with non‐small cell lung cancer and EGFR L858R mutation, efficacy was compared between patients with or without brain metastases.

No significant difference in median PFS existed between patients with or without brain metastases. The median PFS was 18.6 months [95% CI, 15.6–22.8] for patients with brain metastases and 17.1 months [95% CI, 14.1–28.6] for those without brain metastases, exhibiting a *p* value of 0.89 (Figure 4).

### 3.4 PD‐L1 Expression Does Not Correlate With Therapeutic Efficacy

**FIGURE 5:**
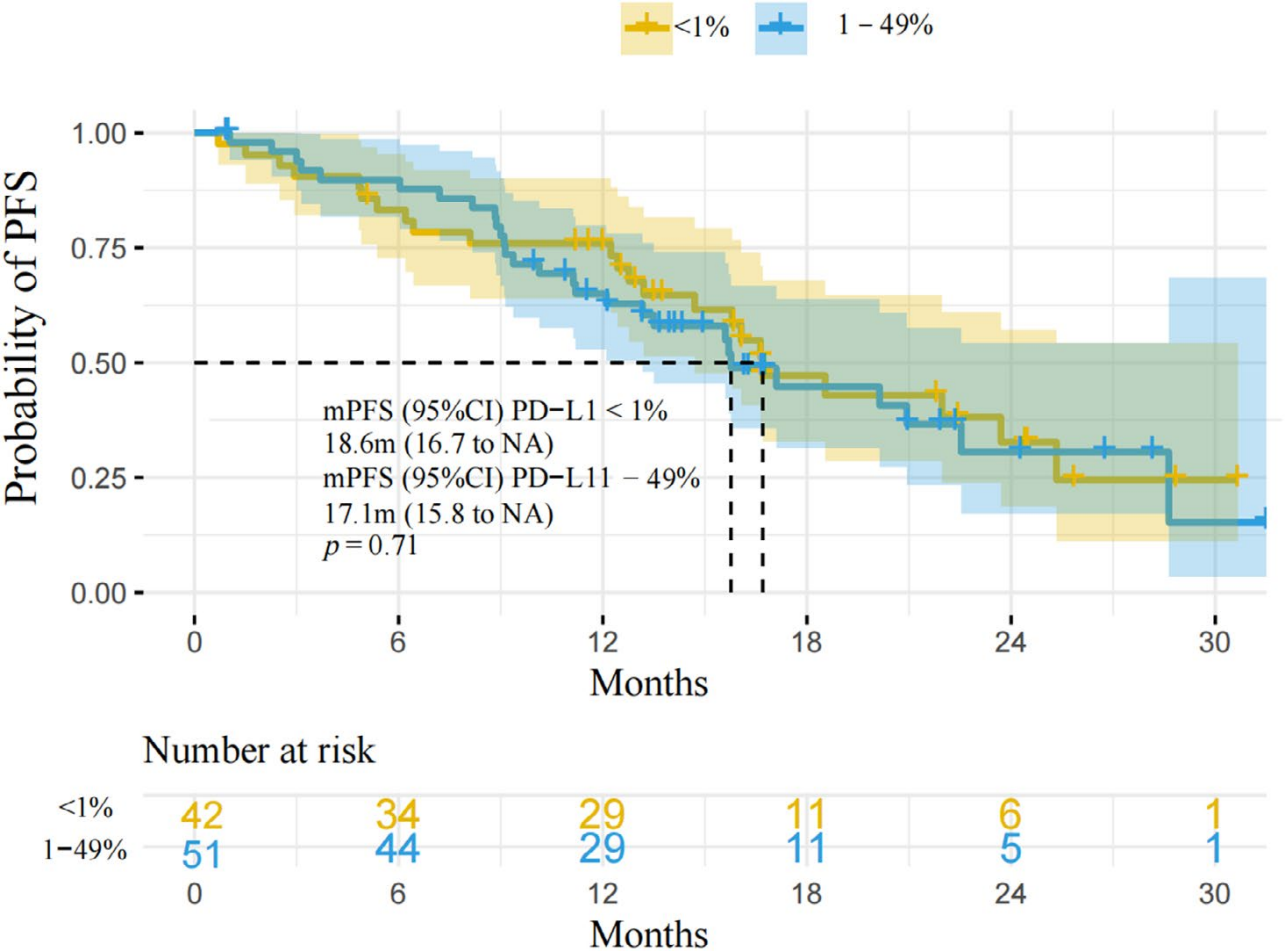
PD‐L1 expression and prognosis. In patients with non‐small cell lung cancer and EGFR L858R mutation, the relationship between PD‐L1 expression and prognosis was examined. PD‐L1, programmed cell death ligand 1.

Patients with PD‐L1 expression between 1%–49% and PD‐L1 expression < 1% showed no significant difference in the median PFS. The median PFS was 16.7 months [95% CI, 14.7–NA] for PD‐L1 expression between 1%–49% and 15.8 months [95% CI, 13.2–NA] for PD‐L1 expression < 1%, with a *p*‐value of 0.71 (Figure 5).

### 3.5 Safety

Both drugs had an overall acceptable safety profile, with most AEs being mild to moderate (Grade 1 or 2). Severe (Grade 3) AEs were rare in both treatment groups, with most AEs reported as non‐Grade 3. Osimertinib had slightly higher rates than almonertinib in most AEs, particularly for rash, diarrhea, leukopenia, and decreased hemoglobin, but the differences were small. Almonertinib had comparable or slightly lower rates of most AEs. Neither treatment was associated with Grade 4 or 5 AEs. The safety and tolerability of osimertinib and almonertinib were comparable, with no significant differences detected (Table 4).

**TABLE 4:** Adverse event between osimertinib and almonertinib.

Adverse event	Osimertinib					Almonertinib					*p*
	Any grade^a^	Grade 1^a^	Grade 2^a^	Grade 3^a^	Grade 4^a^	Any grade^a^	Grade 1^a^	Grade 2^a^	Grade 3^a^	Grade 4^a^	
Diarrhea	38 (31.40%)	34 (28.10%)	4 (3.31%)	0	0	23 (29.11%)	21 (26.58%)	2 (2.53%)	0	0	0.756
Rash	37 (30.58%)	24 (19.83%)	12 (9.92%)	1 (0.83%)	0	26 (32.91%)	16 (20.25%)	10 (12.66%)	0	0	0.757
Decreased white blood cell	34 (28.10%)	27 (22.31%)	7 (5.79%)	0	0	14 (17.72%)	8 (10.13%)	6 (7.59%)	0	0	0.0924
Decreased hemoglobin	28 (23.14%)	22 (18.18%)	6 (4.96%)	0	0	15 (18.99%)	12 (15.19%)	3 (3.80%)	0	0	0.598
Decreased neutrophil	17 (14.05%)	13 (10.74%)	2 (1.65%)	2 (1.65%)	0	9 (11.39%)	4 (5.06%)	5 (6.33%)	0	0	0.835
Decreased albumin	13 (10.74%)	10 (8.26%)	3 (2.48%)	0	0	8 (10.13%)	5 (6.33%)	2 (2.53%)	1 (1.27%)	0	0.999
Decreased platelet	8 (6.61%)	4 (3.31%)	4 (3.31%)	0	0	4 (5.06%)	4 (5.06%)	0	0	0	0.767
Decreased appetite	6 (4.96%)	6 (4.96%)	0	0	0	3 (2)	3 (2)	0	0	0	0.999
Stomatitis	2 (1.65%)	2 (1.65%)	0	0	0	2 (2.53%)	2 (2.53%)	0	0	0	0.648
Pruritus	2 (1.65%)	2 (1.65%)	0	0	0	1 (1.27%)	1 (1.27%)	0	0	0	0.999
Fatigue	1 (0.83%)	1 (0.83%)	0	0	0	1 (1.27%)	1 (1.27%)	0	0	0	0.999

^a^ *n* (%).

## 4 Discussion

To the best of our knowledge, this is the first retrospective study to investigate the therapeutic effects of osimertinib and almonertinib in a single center in patients with advanced NSCLC harboring EGFR L858R mutations. Our results indicate that no significant difference in the mPFS exists between osimertinib and almonertinib. The mPFS was 18.5 months for osimertinib and 19.4 months for almonertinib, with a HR of 0.92. Furthermore, among patients with brain metastases, no significant difference in the mPFS was observed between those treated with osimertinib and those treated with almonertinib. The mPFS was 18.5 months for osimertinib and 19.4 months for almonertinib, with a HR of 0.92. Additionally, forest plots of subgroup analyses showed no significant difference in the median PFS between the almonertinib and osimertinib groups. These results suggest that both almonertinib and osimertinib demonstrate good therapeutic effects against EGFR L858R mutations, with no significant difference in efficacy between the two.

During the FLAURA clinical trial, the mPFS reached 18.9 months in the osimertinib group, compared to 10.2 months in the control group [11, 12]. Our study results showed that the mPFS for osimertinib was 18.5 months. In contrast, real‐world studies reported a median PFS of 18.9 months following a median follow‐up of 12.3 months [6, 24]. Our results are consistent with previous findings demonstrating the effectiveness of osimertinib for treating patients harboring EGFR L858R mutations. Specifically, the mPFS with osimertinib therapy was 19.4 months. Similarly, in the AENEAS study, the mPFS in the almonertinib group was 19.3 months. These results are in line with the clinical trial data. In patients with NSCLC harboring the EGFR‐L858R mutation, no significant difference was observed in the mPFS between almonertinib and osimertinib, exhibiting a HR of 0.92. However, in the FLAURA2 [27] clinical trial, osimertinib combined with chemotherapy demonstrated superior efficacy, with a median PFS of 25.5 months, far exceeding that of osimertinib monotherapy.

In this real‐world retrospective study, EGFR–TKIs were found to have a significant effect on patients with EGFR‐mutant brain metastases. Both almonertinib and osimertinib demonstrated good efficacy in treating brain metastases, with a mPFS of 18.6 months for patients with brain metastases and 17.1 months for those without brain metastases. In another real‐world study including 22 patients, the ORR and disease control rate (DCR) were 40.9% and 86.4%, respectively, with a mPFS of 8.5 months. The median intracranial PFS was not achieved. This analysis further confirmed that osimertinib demonstrates clinically meaningful efficacy against central nervous system metastasis in Chinese patients with advanced NSCLC. Collectively, these results support the notion that EGFR–TKIs have favorable therapeutic effects against brain metastases.

PD‐L1 expression between 1%–49% and PD‐L1 expression < 1% showed no significant difference in the mPFS. The mPFS was 16.7 months for PD‐L1 expression between 1%–49% and 15.8 months for PD‐L1 expression < 1%. Our results indicate that PD‐L1 expression is not associated with EGFR L858R mutation prognosis. These findings were consistent with those of previous studies. A meta‐analysis of six publications showed that positive PD‐L1 expression was not associated with OS or PFS in patients with advanced NSCLC harboring EGFR mutations that received EGFR–TKIs [25]. PD‐L1 expression was neither a predictive nor prognostic factor in these patients [25]. However, these findings are inconsistent with those of previous large‐scale, real‐world studies. For example, in a cohort of patients with EGFR‐mutant lung adenocarcinoma, high PD‐L1 expression was associated with early resistance to first‐generation EGFR‐TKIs and shorter survival, regardless of ethnicity [26]. Another study found that the efficacy of EGFR–TKIs was influenced by baseline PD‐L1 expression. High PD‐L1 expression was associated with shorter PFS. The combined indicators of PD‐L1 have identified subgroups showing divergent benefits from EGFR–TKIs therapy [28]. PD‐L1 TPS ≥ 50% was independently associated with a significantly shorter PFS in the overall population and associated with shorter OS in patients with exon 19 deletion compared with that of PD‐L1 TPS < 50%. Due to the retrospective nature of our study, the sample size with PD‐L1 ≥ 50% was too small to analyze the prognosis. Due to these conflicting results, further research is needed to explore the relationship between PD‐L1 expression and prognosis of EGFR mutations.

This study has some limitations. First, because of the retrospective nature of this study, avoiding selection bias is challenging; therefore, we anticipate more prospective clinical trials in the future. Second, most patients with EGFR L858R mutations did not undergo PD‐L1 testing, and relatively few samples with PD‐L1 levels > 50% were observed. Consequently, comparing the efficacies of almonertinib and osimertinib in this subset was not possible. Further research is needed to compare the effects of almonertinib and osimertinib when PD‐L1 levels ≥ 50%. More studies are also needed to explore the relationship between PD‐L1 expression and the prognosis of EGFR L858R mutations. Third, our sample size was relatively small; thus, future studies should include data from various research centers and larger sample sizes to enhance the reliability and generalizability of our findings. Finally, since our study only evaluated PFS and lacked comparisons of OS, ORR, and DCR, the results suggest a potential similarity in efficacy between almonertinib and osimertinib. In the safety, neither almonertinib nor osimertinib was associated with any Grade 4 or 5 AEs, and the differences between the two agents were minimal. Future studies are still needed to further validate these findings.

## 5 Conclusion

Our study found no significant difference in treatment efficacy between almonertinib and osimertinib in patients with the EGFR L858R mutation. Furthermore, third‐generation EGFR‐TKIs such as almonertinib and osimertinib have shown promising therapeutic effects in the treatment of brain metastases. Additionally, PD‐L1 expression does not affect the prognosis of patients with EGFR L858R mutations. Therefore, both almonertinib and osimertinib are viable options for patients with NSCLC harboring the EGFR L858R mutation based on our findings.

## Author Contributions

Conceptualization: Xiujing Yao. Methodology: Ruyue Li. Validation: Xiujing Yao. Formal analysis: Xiujing Yao. Investigation: Ruyue Li. Data curation: Ying Li, Xiujing Yao, and Xue Dong. Writing – original draft preparation: Xiujing Yao. Writing – review and editing: Xiujing Yao; Ruyue Li. Visualization: Yintao Li. Supervision: Yintao Li.

## Funding

This work was suppoted by the National Natural Science Foundation of China (82373044), the Natural Science Foundation of Shandong Province (ZR2022LSW001) and the Beijing Xisike Clinical Oncology Research Foundation (Y‐2024AZ NSCLC MS‐0100).

## Ethics Statement

This study was approved by the Ethics Committee of Cancer Hospital Affiliated to Shandong First Medical University, which waived the need for informed consent because of the retrospective nature of the study. We declare that the patient's information will be kept confidential and that we adhered to the principles of the Declaration of Helsinki.

## Conflicts of Interest

The authors declare no conflicts of interest.

## Data Availability

The data that support the findings of this study are available from the corresponding author upon reasonable request.
